# High protein ingestion does not affect whole-body insulin sensitivity in individuals with overweight or obesity

**DOI:** 10.1210/jendso/bvag013

**Published:** 2026-01-28

**Authors:** Oana Ancu, Astrid C Hauge-Evans, Fulvia Draicchio, Diana-Elena Neculescu, Ralph Rogers, Nicholas A Burd, Andreas F H Pfeiffer, Martin O Weickert, Nicholas M Hurren, Richard W A Mackenzie

**Affiliations:** School of Life and Health Sciences, University of Roehampton, London SW15 4JD, UK; School of Life and Health Sciences, University of Roehampton, London SW15 4JD, UK; School of Life and Health Sciences, University of Roehampton, London SW15 4JD, UK; School of Life and Health Sciences, University of Roehampton, London SW15 4JD, UK; VisitHealth Medical Clinic, London Institute for Human Performance and Longevity, London W14 0HG, UK; Department of Health and Kinesiology, College of Applied Health Sciences, University of Illinois Urbana-Champaign, Urbana, IL 61820, USA; Department of Endocrinology and Metabolism (Diabetes and Nutritional Medicine), Charité Universitätsmedizin Berlin, Berlin 10117, Germany; German Center for Diabetes Research (DZD), München-Neuherberg 85764, Germany; Department of Clinical Nutrition/DZD, German Institute of Human Nutrition Potsdam-Rehbruecke, Nuthetal 14558, Germany; Warwickshire Institute for the Study of Diabetes, Endocrinology and Metabolism, University Hospitals Coventry and Warwickshire, Coventry CV2 2DX, UK; Division of Biomedical Sciences, Warwick Medical School, Clinical Sciences Research Laboratories, University Hospitals Coventry and Warwickshire, Coventry CV2 2DX, UK; Research Centre for Health & Life Sciences, Faculty of Health & Life Sciences, Coventry University, Coventry CV1 5FB, UK; School of Life and Health Sciences, University of Roehampton, London SW15 4JD, UK; Center for Human Nutrition and Department of Molecular Genetics, University of Texas Southwestern Medical Center, 5323 Harry Hines Blvd, Dallas, TX 75390-9014, USA; School of Life and Health Sciences, University of Roehampton, London SW15 4JD, UK; VisitHealth Medical Clinic, London Institute for Human Performance and Longevity, London W14 0HG, UK; Research Centre for Health & Life Sciences, Faculty of Health & Life Sciences, Coventry University, Coventry CV1 5FB, UK; Coventry University, Institute of Cardio-Metabolic Medicine, University Hospital Coventry & Warwick NHS Trust, Coventry CV1 5FB, UK

**Keywords:** diabetes, high protein, insulin resistance, obesity

## Abstract

**Context:**

High protein diets (HPD), rich in branched-chain amino acids (BCAAs), are proposed to enhance glycemic control. The metabolic implications of elevated BCAAs in insulin resistance (IR) are unclear, but overactivation of ribosomal protein S6 kinase B1 (S6K1)-related signaling pathway may contribute to IR.

**Objective:**

To investigate the impact of 2 dietary protein interventions on IR and molecular signaling in skeletal muscle (acutely) and adipose tissue (18-week period) in overweight/obese individuals.

**Methods:**

The acute study included ingestion of 50 g protein (MPD), 100 g protein (HPD), or 50 g of protein with added fat (MPDAF) on 3 different occasions with muscle biopsies before and after. The 18-week analysis used a subset of data with available adipose tissue biopsies from a randomized, controlled, isoenergetic dietary intervention, focusing on the relevant HPD and control diets. Insulin sensitivity was assessed using labeled intravenous glucose tolerance tests acutely, and by euglycemic-hyperinsulinemic clamp in the 18-week intervention.

**Results:**

Inositol hexakisphosphate kinase 1 (IP6K1) and total AMP-activated protein kinase (AMPK) protein content significantly decreased following the HPD meal (*P* = .048 and *P* = .006 respectively), alongside increased *P*-AktThr^308^/Akt2 (*P* = .046), while S6K1 mRNA was lower after 6 weeks of HPD, compared to the control diet group (*P* = .046), but not at 18 weeks. However, neither intervention changed whole-body IR.

**Conclusion:**

Key proteins implicated in intracellular insulin signaling were altered with an acute HPD meal (decreased IP6K1 and AMPK, increased pAkt/Akt2 activity), indicating potential enhancement of insulin-mediated glucose signaling at the molecular level. These findings suggest that, while systemic IR was unchanged, high protein intake may have beneficial effects on cellular insulin signaling.

New & Noteworthy Diets high in protein are often used as an intervention to manage obesity and associated complications, such as insulin resistance (IR). Yet elevated amino acid availability, using intravenous infusion or in vitro culture models suggest that excessive amino acids may contribute to IR. Our data show that, while there are some molecular changes to suggest dysfunction at the cellular level, this is not extended to our whole-body measures of IR over the short- or medium-term.

Insulin resistance (IR) is the hallmark of metabolic syndrome and type 2 diabetes (T2D) and can be influenced by dietary interventions causing weight loss [1]. Various diet models have been proposed to manage glycemic control, but high protein diets (HPDs), rich in branched-chain amino acids (BCAAs; ie, isoleucine, leucine, and valine) are commonly recommended [2]. The relationship between high concentrations of circulating BCAAs and IR remains controversial. Elevated BCAAs concentrations may contribute to IR or could be a consequence of impaired insulin action and not a direct consequence of HPDs. Yet, elevated levels of plasma BCAAs have been linked to IR and could be a predictor of the development of T2D up to 12 years beforehand [3-6].

BCAAs are potent activators of the protein synthesis pathway in peripheral tissue via the activation of mechanistic target of rapamycin complex 1 (mTORC1), in both resting and post-exercised skeletal muscle [7]. mTORC1 is a serine-threonine protein kinase that acts as a crucial regulator of cell growth and metabolism, including lipid and protein synthesis, energy storage, and mitochondrial biogenesis, and dysregulation of this pathway has been implicated in numerous diseases, including cancer and metabolic syndrome [8]. In the presence of persistent hyperaminoacidemia, overactivation of mTORC1 leads to the phosphorylation of ribosomal protein S6 kinase 1 (S6K1) and the subsequent deactivation of phosphatidylinositol 3-kinase (PI3K) [9]. PI3K has an essential role in insulin-dependent glucose uptake via the activation of Akt, which promotes AS160-associated glucose transporter-4 (GLUT-4) translocation. This negative feedback loop on PI3K/Akt/AS160 can result in decreased GLUT-4 translocation and glucose uptake, contributing to the pathogenesis of IR and T2D [10].

While acute hyperaminoacidemia is generally considered safe in healthy individuals, it can have various consequences in individuals with obesity, particularly when coupled with IR and other metabolic dysfunctions commonly observed in obesity. An investigation using obese Zucker rodents showed that hyperaminoacidemia impaired glucose tolerance, decreased insulin sensitivity, and increased hepatic glucose production (HGP) compared to control rats [11]. In vivo human studies also show a decrease in insulin sensitivity during amino acid infusions, especially BCAA [12, 13]. However, considering that intravenous amino acid infusion bypasses the digestive system and the complex hormonal and neural signals involved in nutrient sensing and metabolic regulation that occur during normal dietary protein digestion, and additionally greatly exceed levels observed after oral protein intake, the metabolic responses observed with intravenous amino acid infusion may not fully reflect the dietary protein intake.

We aimed to investigate the effects of moderate and high protein meals, acutely, on both whole-body glucose metabolism and molecular markers implicated in insulin-stimulated glucose uptake in skeletal muscle in individuals with obesity. Comparisons were made between the trials to assess if the high protein meal induced intracellular dysregulation causing a downregulation in insulin signaling when compared with the moderate dose or a controlled meal. To provide additional metabolic insights on the impact of protein intake, we conducted further analysis on cellular signaling events in adipose tissue from a previously published 18-week controlled interventional study in overweight or obese participants [14], where protein intakes were manipulated.

## Methods

### Study design

The acute study employed a randomized order, double-blinded design with a 14-day washout period with 3 conditions differentiated by a single meal provided (Fig. S1; Figshare [15]). The sample size was calculated based on insulin sensitivity data from our previous work using similar methods and populations (prediabetes and T2D) [16-18]. Participants had either a chicken meal containing 50 grams of protein (moderate protein dose [MPD]), 100 grams of protein (high protein dose HPD]) or 50 grams of protein with added fat (moderate protein dose and added fat [MPDAF]) to match the energy content of the HPD meal to investigate whether differences with the higher protein dose were dietary protein-specific or were attributable to the energy content of the meal. Participants were advised to spend approximately 15 minutes to consume the meal without being required to eat quickly.

In the medium-term study, participants were randomly assigned to an 18-week isoenergetic nutritional intervention in 111 overweight participants with features of the metabolic syndrome, divided into balanced groups (Table S1; Figshare [15]). Details of that dietary intervention have been published [5, 14, 19]. Diets were kept isoenergetic with identical dietary fat contents in all dietary groups and varying dietary protein and carbohydrate contents. Adipose tissue biopsies were performed in a subset (HPD group, n = 7 [6 female, 1 male]; control group, n = 6 [5 female, 1 male]), sampled after 0, 6, and 18 weeks of dietary intervention. Gene expression data of that study have not been published previously.

### Ethical approval and participant recruitment

Ethical approval for the acute experimental design and procedures was granted by the University of Roehampton Ethics Committee (reference number LSC 189/235) prior to the start of data collection. Participants were recruited through advertisements placed locally and online and through university-wide group emails. Participants for the medium-term study were recruited from the Metabolic-Syndrome-Berlin-Potsdam Study cohort (∼n = 2700) [20]. The Ethics Committee of the University of Potsdam approved the study (BMBF FKZ 0313826). All experimental procedures were conducted in accordance with the World Medical Association's revised declaration of Helsinki for Medical Research Involving Humans [21] and written informed consent was obtained from all participants.

### Inclusion/exclusion criteria for participants

To qualify for inclusion in the acute study, individuals needed to meet the following criteria: a fasting blood glucose concentration below 7 mmol/L (<126 mg/dL), indicating glucose levels within the nondiabetic range; a body mass index (BMI) equal to or greater than 30 kg/m^2^; and an age range of 30 to 65 years.

Exclusion criteria included: pregnant women or those taking hormone contraception therapy; individuals with uncontrolled hypertension or secondary complications of metabolic syndrome (such as neuropathy, nephropathy, cardiovascular diseases or stroke); individuals with a known diagnosis of active cancer; current smokers; individuals requiring insulin or any other glycemia-altering medication; individuals diagnosed with arthritis, rheumatism, or gout spondylitis; and individuals unable to mobilize independently. Details about inclusion and exclusion criteria in the medium-term study have been published [14].

Nine individuals with obesity (mean age 46.1 ± 9.7 years, body mass 94.9 ± 9.3 kg, BMI 33.2 ± 2.0 kg/m^2^, body fat 43.5% ± 10.2%, fasting blood glucose 4.5 ± 0.4 mmol/L, HbA1c 36 ± 1 mmol/mol n = 5 male, n = 4 female) were recruited for participation in the acute study and attended the laboratories at University of Roehampton on 4 different occasions. Thirteen participants (control group = 6, HPD group = 7) were included in the medium-term study analysis (mean age, control group 57.1 ± 2.4 years and HPD 54.8 ± 2.9 years; body mass, control group 83.9 ± 4.2 kg and 86.4 ± 5.1 kg HPD; BMI, control group 30.6 ± 1.0 kg/m^2^ and HPD group 32.5 ± 1.3 kg/m^2^.

### Experimental protocol

Participants attended the laboratory for the experimental protocol at ∼ 9:00 Am on 3 occasions, having fasted for 12 hours prior, with each visit separated by 14 to 30 days. To allow for administration of glucose solution during the intravenous glucose tolerance test (IVGTT), a cannula was placed into the antecubital vein of one arm. A separate cannula was placed into a dorsal hand vein, in a retrograde direction, for frequent sampling of arterialized-venous blood, achieved through use of a thermoregulated hot box (∼ 60 °C) as previously described [17, 22].

Muscle biopsies were collected under local anesthesia from the vastus lateralis with the conchotome method [23] at baseline (0 minutes). Immediately following the baseline blood sample and muscle biopsy, participants were provided a chicken meal containing 50 grams of protein (MPD), 100 grams of protein (HPD), or 50 grams of protein with added fat to match the energy content of the HPD meal (MPDAF). The fat that was added in the MPDAF consisted of 25 g of vegetable oil, which was the equivalent of 231 kcal. The software Dietplan 7 (Forestfield Software Ltd, UK) was used for food composition analysis (Table 1).

**Table 1:** Meal composition analysis for the 3 trials completed in the acute study

Trial	Protein (g)	Chicken (g)	Added fat (g)	Energy (kcal)
MPD	50	156	0	231
HPD	100	312	0	462
MPDAF	50	156	25	462

Abbreviations: HPD, high protein dose; MPD, moderate protein dose; MPDAF, moderate protein dose and added fat.

Following the meal, a 4-hour labeled IVGTT was administered (28.4 mg/kg [6,6-^2^H_2_]glucose and 250 mg/kg unlabeled glucose), prepared under sterile conditions. Thereafter, frequent arterialized (∼5 mL) blood samples were drawn over the ensuing 240 minutes, as previously described [17].

A second muscle biopsy was collected immediately after the IVGTT and ∼240 minutes following the finish of trial meals. Muscle samples were washed in ice-cold saline immediately before being flash frozen in liquid nitrogen and transferred to −80 °C until analysis [23]. A 5 µL sample was used to determine glucose concentrations (Biosen C-Line, EKF Diagnostics, UK) before the remaining samples were centrifuged at 4 °C, 6000 rpm (2000*g*), for 10 minutes. The resulting plasma was aliquoted (1.5 mL) and stored at −80 °C until further analysis. Details related to euglycemic-hyperinsulinemic clamp (EHC) and adipose tissue sampling/preparation in the medium-term study have been published [14].

### Controlled meal preparation

All meals for the acute study were prepared at the University of Roehampton, Whitelands College in the Food Laboratory. The meals consisted of baked chicken which was prepared on the same day of the trial. The chicken was baked in the oven at 180 °C for 30 minutes and blended before served. For the MPDAF trial (50 grams of protein plus fat added to match the energy content of the high protein trial), vegetable oil was added to the blended chicken until the energy content of the HPD meal was matched. Details related to the dietary contents and supplements used in the medium-term study have been published [14]

### Blood analysis

Insulin concentrations were determined in plasma using a commercially available ELISA (EIA-2935, RRID:AB_2891339, DRG Instruments GmbH, Germany). Blood glucose concentrations were determined using Biosen C-Line (EKF Diagnostics, UK). Labeled glucose was measured in plasma by gas chromatography–mass spectrometry as previously described [18].

### Proton magnetic resonance spectroscopy for the measurement of hepatic lipid contents

Proton magnetic resonance spectroscopy (^1^H-MRS) for the measurement of hepatic lipid content was performed as described [24]. For details, see Online Supporting Materials under “Supplemental data” in the online version of the original paper [14].

### Data modeling

Plasma insulin, glucose concentrations, and [6,6-^2^H_2_]glucose enrichments values were used to model the metabolic indices: insulin sensitivity (S_I_^2*^), glucose effectiveness (S_G_^2*^), and hepatic glucose production (HGP) using 2-compartment modeling, as described previously [18, 25, 26] (SAAMII Institute, Seattle, WA).

The incremental area under the curve (iAUC) of IVGTT was calculated for insulin and glucose concentrations as described in [27]. Additionally, the acute insulin response to glucose (AIRg) was calculated using the iAUC of insulin concentration in the first 10 minutes, while second-phase insulin secretion was determined from 11 to 240 minutes of the IVGTT using the trapezoidal rule. Disposition index (DI = S_I_^2*^×AIRg) was calculated as described in [28].

To assess whole-body insulin sensitivity, the medium-term duration study employed a euglycemic-hyperinsulinemic clamp (EHC) at baseline (week 0), and after 6 and 18 weeks for each of the dietary interventions [14].

### Muscle analysis

Muscle samples were lysed and analyzed via Western blot for protein analysis as described [18]. The postintervention muscle biopsy data were normalized to the average baseline expression across the 3 experimental conditions for each individual to account for individual variability in baseline levels. The primary antibodies used were: anti-total Akt2 (#3063, RRID:AB_2225186) [29], pAkt^S473^ (#9271, RRID:AB_329825), pAkt^T308^ (#9275, RRID:AB_329828), pS6K1Th^r389^ (#9234, RRID:AB_2269803), pAMPKα1^Ser485^/AMPKα2^Ser491^ (#4185, RRID:AB_2169402), anti-AMPKα (#2532, RRID:AB_330331) (all Cell Signaling Technology), Anti-IP6K1 antibody (ab129595, RRID:AB_11157733) and recombinant anti-S6K1 (ab32359, RRID:AB_777802) (Abcam). Signals observed after the meal was normalized to the respective participant's average baseline signal.

### RNA extraction from subcutaneous adipose tissue

Fat tissue biopsies were performed as previously described [14]. Total RNA was extracted from 500 mg frozen subcutaneous fat tissue homogenates in Qiazol™ followed by use of Lipid Tissue RNA-Kit™ (Qiagen, Germany) according to the manufacturer's protocol. The samples were stored at −80 °C until single-stranded cDNA synthesis. Quantitative real time polymerase chain reaction (qRT-PCR) was performed for several genes in human adipose tissue. From 1 µg of RNA samples single-stranded cDNA was synthesized using the High-Capacity cDNA Reverse Transcription Kit™ with random priming (Applied Biosystems, Germany). cDNA sample concentrations were 0.5 pg/µL in the qRT-PCR assays, carried out in optical 384-well plates and labeled by Power SYBR Green™ master mix (Applied Biosystems, Germany). All samples were determined as triplicates, and non-template controls were measured. The mixture was incubated at 95 °C for 10 minutes and then cycled at 95 °C for 15 seconds and at 60 °C for 1 minute for 47 times using the ABI Prism 7900HT Sequence Detector (Applied Biosystems, Germany). The amplification efficiencies of each primer pair were tested in cDNA dilution series (r2 > 0.8). In every gene expression assay a cDNA dilution series was present for each primer pair used (standard curve method). RNA was reverse transcribed into cDNA using the High-Capacity cDNA Reverse Transcription Kit™ with random priming (Applied Biosystems, Germany). cDNA was labeled by Power SYBR Green™ master mix (Applied Biosystems, Germany) and analyzed in an ABI Prism 7900HT Sequence Detector (Applied Biosystems, Germany) to determine mRNA levels of the following: mTOR (5′ CTTGCGTTGGAACATCCAAAGT-3′, reverse 5′-TCATTGGAGACGGTTTGGTGA-3′); IRS1 (5′-CGAAAGAGAACTCACTCGGCA-3′, reverse 5′-TAGGCAGGCATCATCTCTGTGT-3′); 4EBP1 (5′-ACCCGATGACGCACAATTTG-3′, reverse 5′-TTTGGATGCCCCAGGAAGA-3′); p70S6K (5′-TGGCATGGAACATTGTGAGA-3′, reverse 5′-TAGCCCCCTTTACCAAGTACCC-3′); Akt (5′-AGCGACGTGGCTATTGTGAAG-3′, reverse 5′-CTGCGCCACAGAGAAGTTGTT-3′); PPARγ (5′-CGAGGGCGATCTTGACAG-3′, reverse 5′-TCTTTGCTCTGCTCCTGC-3′). For quantification, the standard curve method was applied. Target genes were normalized to relative expression levels of ribosomal protein large protein 0 (RPLP0) which is an established housekeeping gene in adipose tissue. Fold changes were calculated from the ratio of means of the normalized quantities and their statistical significance was determined by unpaired Student's *t* test.

### Statistical analyses

Statistical analyses were carried out using IBM SPSS Statistics (version 28.0.1.1.). In the acute study, differences between the conditions in muscle protein content were evaluated using ANOVA, with Bonferroni correction applied to account for multiple comparisons., or two-tailed Student's *t* test for paired sample analyses. Data from the medium-term study were analyzed using a 2-way repeated measures ANOVA (2 × 3, group by time) to assess differences between baseline, 6 weeks, and 18 weeks with Bonferroni correction applied for post hoc pairwise comparisons. Data are expressed as mean (standard error of the mean [SEM]), unless otherwise specified. Statistical significance was accepted at *P* < .05.

## Results

### Acute study

**Figure 1:**
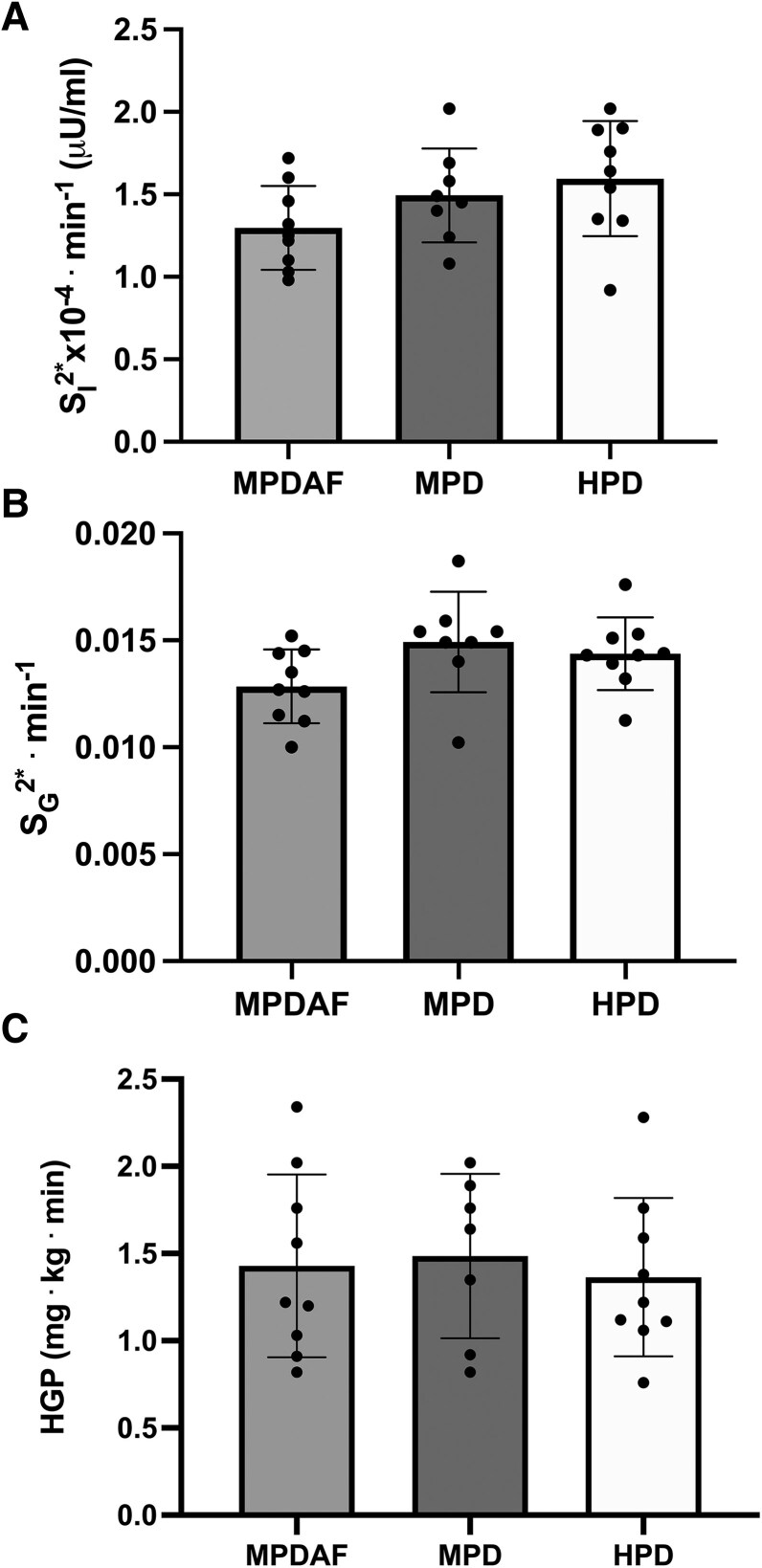
Insulin sensitivity (S_I_^2*^) (A), glucose effectiveness (S_G_^2*^) (B) and hepatic glucose production (HGP) (C) calculated for each of the trials: MPDAF, MPD, and HPD (n = 9 for each group). Data expressed as mean ± SEM. No significant differences between trials S_I_^2*^, *P* = .14; S_G_^2*^, *P* = .11; HGP, *P* = .88. Abbreviations: HPD, high protein dose; MPD, moderate protein dose; MPDAF, moderate protein dose and added fat.

Two-compartment modeling of the IVGTTs showed no significant differences between meals (MPDAF, MPD, and HPD) for insulin sensitivity (S_I_^2*^) (*P* = .14), glucose effectiveness (S_G_^2*^) (*P* = .11) or HGP (*P* = .88) (Fig. 1).

**Figure 2:**
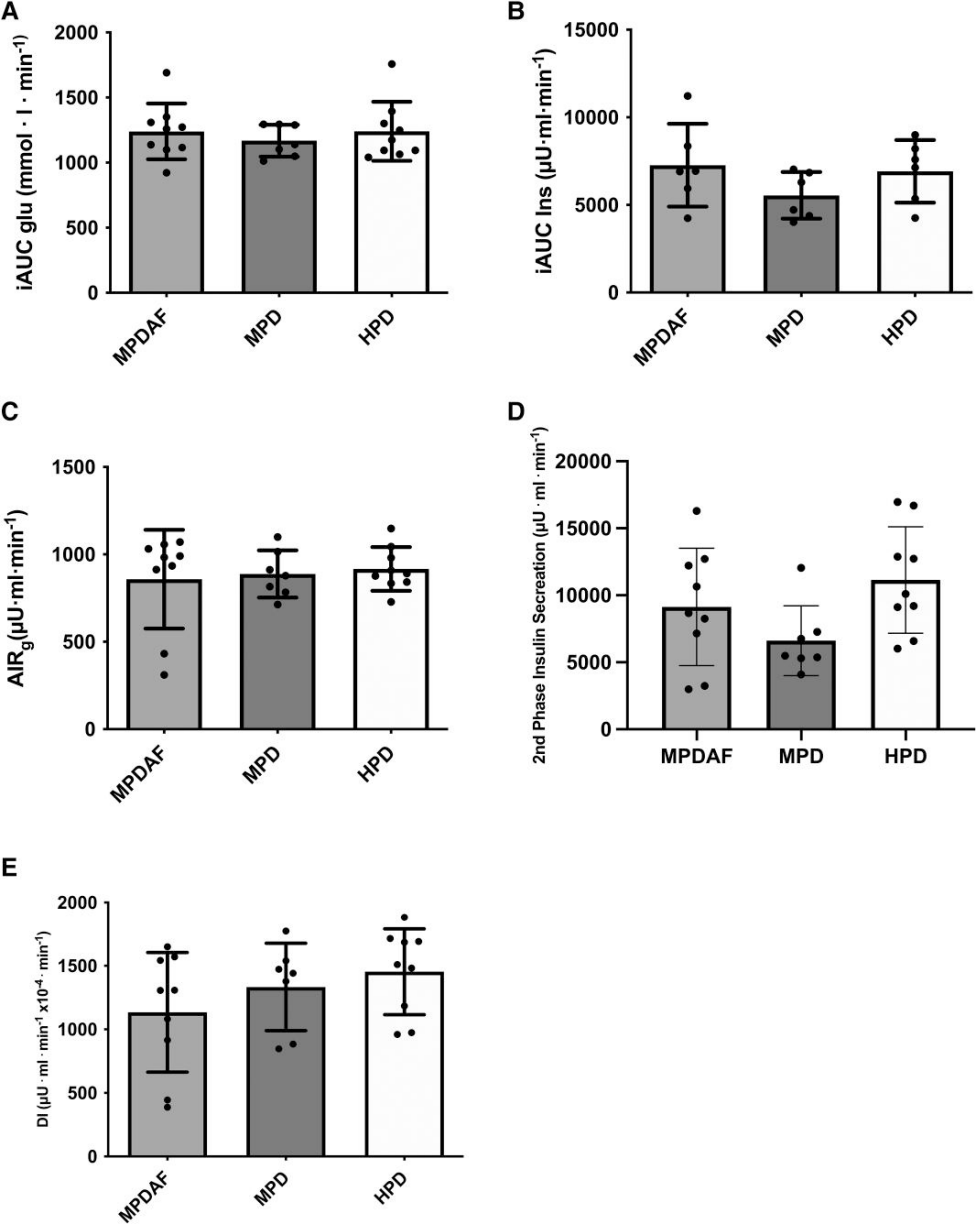
Incremental area under the curve for glucose (iAUC_glu_) concentration during IVGTT (A); iAUC_Ins_ concentrations during IVGTT (B); acute insulin response to glucose (AIR_g_) (C); second-phase insulin secretion (D); and disposition index (DI) (E); n = 9 for each group. Data expressed as mean ± SEM. Abbreviations: HPD, high protein dose (100 g); MPD, moderate protein dose (50 g); MPDAF, moderate protein dose added fat (50 g protein and 25 g fat).

From plasma analysis and further calculations, this study found no statistical differences between the 3 conditions (HPD, MPD, and MPDAF) for iAUC_glu_ (*P* = .72), iAUC_ins_ (*P* = .01), AIRg (*P* = .82), second-phase insulin secretion (*P* = .11), disposition index (DI) (*P* = .24) and DI second-phase insulin secretion (*P* = .08) (Fig. 2). Plasma amino acids data for the IVGTT study is available in Fig. S2 and S3 [15], showing an increase in some BCAA over time. More specifically, within the HPD trial valine concentrations significantly increased from baseline from minute 60 after meal ingestion and have been maintained significantly increased for the remaining duration of the trial (up to 4 hours). In a similar manner, valine concentrations were also increased from baseline in the MPD trial from minute 60 until 120 minutes post meal ingestion. Within the MPDAF trial, we saw no significant changes compared to baseline at any time point following meal ingestion. In addition, valine's plasma concentration was significantly higher in the HPD trial compared to MPD and MPDAF at 180 minutes post meal (*P* = .032 and .012 respectively) and different compared to MPDAF at 240 minutes post meal (*P* = .029).

Leucine concentrations were significantly higher compared to baseline from minute 60 to minute 180 after meal ingestion within the HPD, whereas in the MPD trial they were significantly higher than baseline only at minute 60 and 80 after meal ingestion, with no significant increases noted in the MPDAF trial. Additionally, leucine concentrations were higher in the HPD trial when compared to the other 2 conditions (MPDAF and MPD) at 180 minutes post meal ingestion (*P* = .015 and *P* = .031 respectively).

While no changes were seen in isoleucine concentrations over time after the meal or between the conditions (MPDAF, MPD, and HPD) at any time point (30-240 minutes), total BCAA concentration was significantly higher compared to baseline from minute 80 to minute 180 post meal ingestion in the HPD trial, whereas in the MPD it was increased compared to baseline from minute 60 to minute 80, with no significant changes compared to baseline seen in the MPDAF. In addition, total BCAA concentrations in the HPD trial was significantly higher when compared to MPDAF concentrations at 180 minutes post meal ingestion (*P* = .035) (Fig. S2 [15]).

**Figure 3:**
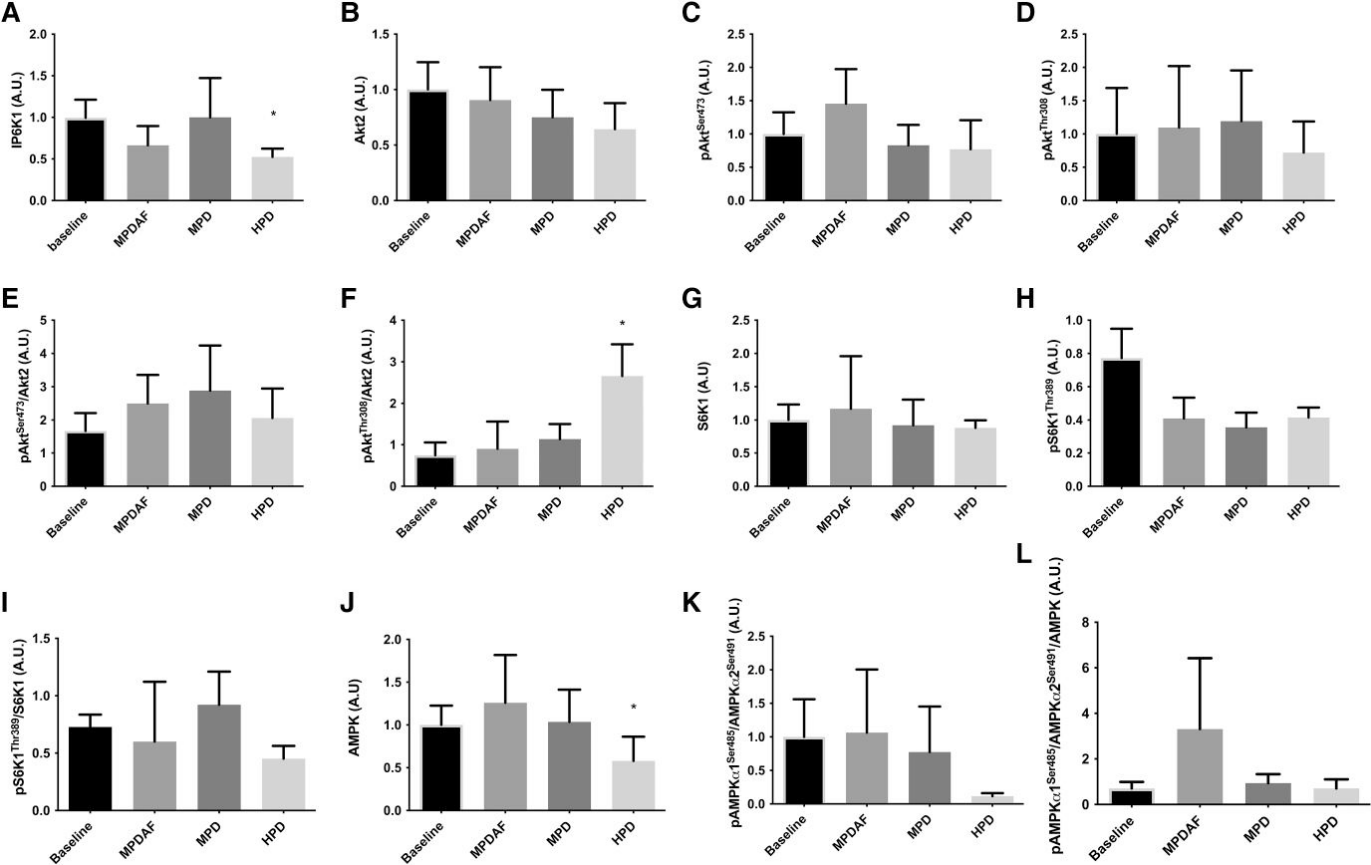
Skeletal muscle protein content for IP6K1 (A), Akt2 (B), pAkt^Ser473^(C), pAkt^Thr308^ (D), pAkt^Ser473^/Akt2 (E), pAkt^Thr308^/total Akt2 (F), total S6K1 (G), pS6K1Th^r389^ (H), pS6K1Th^r389^/total S6K1 (I), total AMPK (J), pAMPKα1^Ser485^/AMPKα2^Ser491^ (K), and pAMPK_α1_^Ser485^/AMPK_α2_^Ser491^/total AMPK (L) (baseline n = 9, MPDAF n = 8, MPD n = 7, HPD n = 8). Data expressed as mean ± SEM. *denotes significant differences from baseline, *P* < .05. Abbreviations: HPD, high protein dose; MPD, moderate protein dose; MPDAF, moderate protein dose and added fat.

From Western blot analysis, protein content of IP6K1 (Fig. 3A) decreased in the 4 hours following the HPD when compared with baseline (*P* = .048), with no significant differences in the MPD (*P* = .89), nor in the MPDAF (*P* = .33) for the same comparison (ie, against averaged baselines within trial). The level of pAkt^Ser308^/Akt2 (Fig. 3F) increased from baseline to the 4-hour sample in the HPD trial (*P* = .046). Finally, AMPK (Fig. 3J) decreased from baseline in the HPD trial (*P* = .01), with no significant differences between the groups. No significant differences were observed between the conditions for Akt2 (Fig. 3B), pAkt^Ser473^(Fig. 3C), pAkt^Thr308^ (Fig. 3D), pAkt^Ser473^/total Akt2 (Fig. 3E), total S6K1 (Fig. 3G), pS6K1Th^r389^ (Fig. 3H), pS6K1Th^r389^/total S6K1(Fig. 3I), pAMPK_α1_^Ser485^/AMPK_α2_^Ser491^ (Fig. 3K), and (pAMPK_α1_^Ser485^/AMPK_α2_^Ser491^)/total AMPK (Fig. 3L).

### Medium-term study

**Figure 4:**
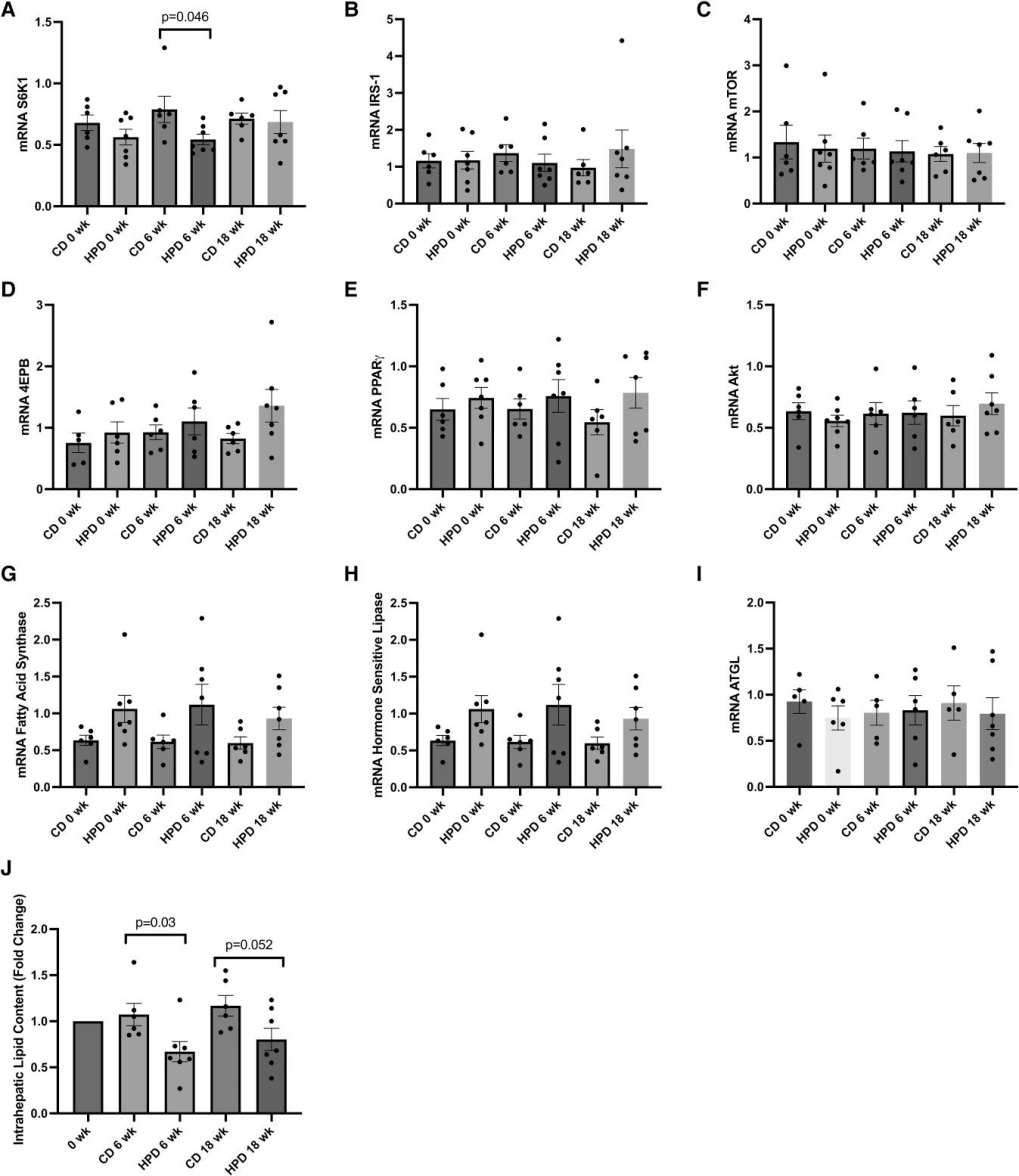
Adipose tissue mRNA expression for S6K1 (A), IRS-1 (B), mTOR (C), 4EPB (D), PPARγ (E), Akt (F), fatty acid synthase (G), hormone-sensitive lipase (H), ATGL (I), and intrahepatic lipid content (J) (fold change from baseline) at baseline, 6 weeks, and 18 weeks following a control diet (CD; n = 6) and high protein diet (HPD; n = 7). *Significant difference between groups at 6 weeks for S6K1 (*P* < .05).

**Figure 5:**
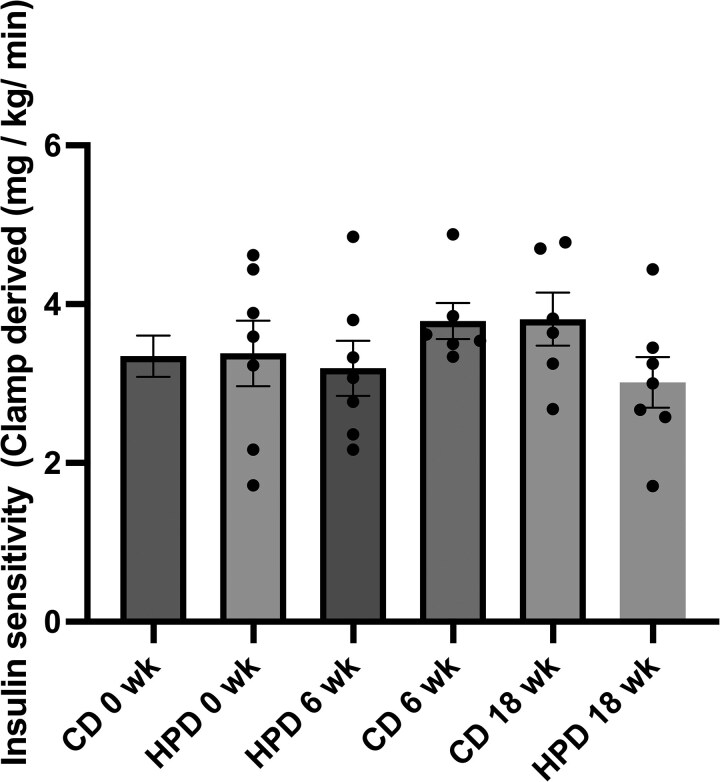
Insulin sensitivity at baseline, 6 weeks, and 18 weeks following a control diet (CD; n = 6) and high protein diet (HPD; n = 7). No significant differences were noted.

The medium-term study revealed a significant difference in the mRNA levels of S6KI between HP and control diet at 6 weeks (*P* = .046), which was not witnessed at 18 weeks for the same group comparison (*P* = .80). No significant differences were noted for mRNA expression for IRS-1, mTOR, 4E-BP, PPARγ, Akt, fatty acid synthase, hormone-sensitive lipase, or adipose triglyceride lipase (ATGL) between baseline, 6 weeks and 18 weeks following both the control diet and high protein diet (HPD) (Fig. 4). There was a significant difference observed for intrahepatic lipid content (IHL), which was significantly lower in the HP diet group at 6 weeks compared to control diet (*P* = .03). The same comparison at 18 weeks showed a trend toward significance (*P* = .052) (Fig. 4J). In addition, no changes in insulin sensitivity between groups at 6 or 18 weeks were seen, as measured using the EHC (Fig. 5). In the medium-term study, all diets were designed to be isoenergetic and there was no change in body weight in any of the dietary groups between baseline (week 0) and later time points including the 6-week time point (*P* = .42). Dietary amino acid signature data for the medium-term study have been previously published by Hattersley and colleagues [5].

## Discussion

Our acute study investigated the effects of different protein doses on the cellular mechanisms that are central in the regulation of insulin-stimulated glucose uptake and protein synthesis in skeletal muscle in individuals with obesity. For the first time in humans, this study employed the 2-compartment IVGTT to assess if the different meals affected whole-body insulin sensitivity. Despite variations in protein intake, the current data set showed no statistically significant differences for insulin sensitivity, glucose effectiveness, or HGP between conditions (HPD, MPD, and MPDAF). These findings are, of course, following a short-term dietary manipulation. We acknowledge that the use of the labeled IVGTT resulted in our data representing insulin secretion in response to both protein ingestion and intravenous glucose. However, as the IVGTT glucose load remained fixed for each volunteer and for each of their trials, we were able to isolate the insulin response to the protein meal.

We have included additional analyses of a similar dietary intervention over an 18-week period [14]. Employing a euglycemic-hyperinsulinemic clamp (EHC), these data from in a subgroup of participants with available adipose tissue biopsies showed no differences between high protein and control diets on whole-body insulin sensitivity. We hypothesized that the higher protein ingestion would result in hyperaminoacidemia, leading to an increase in phosphorylation of S6K1 [9] and a subsequent decrease in insulin signaling at the molecular level. While we did observe an increase in amino acid concentration in the HPD trial in the acute study (Figs. S2 and S3 [15]), there were no observed differences in muscle pS6K1Th^r389^ for the same comparisons. In addition, S6K1 mRNA was significantly lower in the high protein compared to control, as measured in adipose tissue, at 6 weeks.

Tremblay and colleagues used an EHC coupled with amino acid infusions leading to an approximately 2.5-fold increase in blood amino acid concentration and a 3.7-fold elevation in skeletal muscle S6K1 protein content [10]. Building upon these insights, the present studies sought to examine the alterations in muscle S6K1 protein content 4 hours following the consumption of different sized protein meals, and over 18 weeks of HPD, hereby providing insights into the physiological dynamics of S6K1 postprandially. No significant changes were observed in either the total S6K1 protein content or its phosphorylated state at Thr^389^ during the acute study for any of the conditions, indicating that the influence of plasma amino acid levels on S6K1 may be notably attenuated compared to infusion studies. However, the medium-term study showed a decrease in S6K1 mRNA expression in adipose tissue over the strictly controlled 6-week period with HPD when compared to an isoenergetic control diet (*P* = .046), whereas after 18 weeks, no significant differences were seen, which is in agreement with the findings observed in the entire cohort [14] and likely related to an observed drop in the adherence to the HPD in the medium-term; as was supported by measurement of biomarkers of protein intake and entirely expected in medium-term dietary interventions in humans that does not include a fully controlled feeding intervention. These findings may suggest that medium-term high protein feeding is required to manipulate S6K1 and that this response may be tissue specific. Nevertheless, researchers noted an increased pS6K1 at Thr^389^ in skeletal muscle induced by elevated insulin, as seen when performing EHC studies [10]. The conflicting results regarding this signaling kinase may be attributed to variations in insulin response between studies. In our current dataset, no significant changes in insulin response were observed, which could explain the absence of differences in this signaling kinase between trials.

It is worth noting that in the published work of Weickert (2011) there was a significant worsening in insulin sensitivity after 6 weeks of a HPD in a larger sample size (n = 23; 4.20 +/− 0.38 (0 week) to 3.71 +/− 0.36 mg·k min^−1^ [6 weeks], *P* = .13) [14]. The effect observed in the sample with available gene expression data used in this study (control n = 6; HPD n = 7) agrees with observations in obese subjects with moderate T2D [30, 31]. These authors showed a similar decrease of hepatic fat on high protein diets after 6 weeks by about 40%, and no changes of fasting amino acid levels or of the insulin signaling pathway [30, 31].

We also observed a decrease in muscle IP6K1 protein content in the acute study in the HPD trial when compared to baseline fasting samples. These results suggest that acutely, 4 hours post meal ingestion, IP6K1 content was downregulated by dietary protein intake only after a certain intake threshold, as no differences were seen in the MPD trial, or when energy intake was matched in the MPDAF. The regulation of IP6K1 in skeletal muscle is of interest due to its role in the modulation of glucose metabolism and insulin signaling [18, 32-35]. An inhibition or genetic deletion of IP6K1 was shown to protect mice from hepatic steatosis by improving mitochondrial function and reducing gluconeogenic pathways while reducing lipolysis in adipose tissue [32]. The inhibition of IP6K1 positively regulates numerous metabolic pathways and may therefore explain the positive effects of high protein diets observed in humans with metabolic dysfunction [36].

Our acute data set also showed an increase in Akt2 activity, in agreement with previous publications [18, 34, 37-39]. The heightened availability of BCAAs, particularly leucine, subsequent to high protein meals, is acknowledged for its potential to activate mTOR and its downstream targets, including S6K1. In some in vitro studies, such activation has been linked to reduced insulin action on Akt, thereby impairing insulin signaling [40, 41]; and supported by previous findings where amino acid infusion caused a rise in IR and S6K1 activity [10]. However, over the 18-week study, we did not observe significant changes in adipose tissue total Akt and pAkt, or Akt mRNA. Although we noted significantly elevated levels of BCAA acutely, over the 4 hours post meal, following the HPD trial, which outlasted those seen at lower doses of protein (MPDAF and MPD), substantial alterations in S6K1 were not seen acutely. Consequently, distinctly from the intravenous infusions studies where the digestion system is circumvented and unphysiologically high levels of amino acids are achieved [10], in the meal-induced context, the increased in pAkt^Thr308^/Akt2 seen following the HPD meal could potentially be attributed to IP6K1 modulation rather than S6K1 activation, although the relatively small sample size in our studies is acknowledged. Additionally, our results may have missed important changes in insulin signaling owing to the time of the second muscle biopsy. The changes in IP6K1 muscle content are supported by a previous study that also noted Akt activation in response to high protein intake correlated with change in IP6K1 content [42].

Furthermore, we had previously published effects of isoenergetic HPD vs control diet with comparable dietary fat contents on intrahepatic lipid (IHL) over an up to 18-week period [14]. In the currently analyzed subset of participants with available gene expression data, we observed a significant decrease in IHL in the HPD group compared to the control diet after the 6-week time point, with a trend still observed after 18 weeks. These findings are consistent with the work of Xu et al [43], who reported a significant 42.6% reduction in IHL among morbidly obese individuals following a calorie-restricted, isocaloric HPD, compared to a low protein diet, and as described by Markova et al upon intake of isocaloric high protein diets in patients with T2D [31]. Further, in the published data from the larger cohort [14], the drop in IHL from baseline following 6 weeks of isoenergetic HPD vs control diet happened despite unchanged body mass in all dietary groups, thereby excluding any weight-change related effects of the diets.

### Conclusion

In conclusion, our findings contribute explaining the potential of high protein meals as a dietary intervention for enhancing metabolic health and glycemic control. While none of the meals tested (MPD, MPDAF, or HPD) induced significant alterations in insulin sensitivity or glucose effectiveness using a labeled IVGTT, the consumption of a high protein meal (containing 100 grams of protein) resulted in a notable reduction in muscle IP6K1 levels and an increase in Akt activity, suggesting that protein consumption can improve insulin signaling, in an acute context.

## Data Availability

The datasets generated and/or analyzed during the current study are not publicly available due to privacy restrictions and ongoing analyses but are available from the corresponding author on reasonable request.

## Abbreviations

AIRg: acute insulin response to glucose
ATGL: adipose triglyceride lipase
BCAA: branched-chain amino acid
BMI: body mass index
DI: disposition index
EHC: euglycemic-hyperinsulinemic clamp
GLUT-4: glucose transporter-4
HGP: hepatic glucose production
HPD: high protein diet
iAUC: incremental area under the curve
IHL: intrahepatic lipid
IR: insulin resistance
IVGTT: intravenous glucose tolerance test
MPD: moderate protein dose
MPDAF: moderate protein dose and added fat
mTORC1: mechanistic target of rapamycin complex 1
PI3K: phosphatidylinositol 3-kinase
S6K1: ribosomal protein S6 kinase 1
T2D: type 2 diabetes
